# Visible and infrared three-wavelength modulated multi-directional actuators

**DOI:** 10.1038/s41467-019-12583-x

**Published:** 2019-10-04

**Authors:** Bo Zuo, Meng Wang, Bao-Ping Lin, Hong Yang

**Affiliations:** School of Chemistry and Chemical Engineering, Jiangsu Province Hi-Tech Key Laboratory for Bio-medical Research, State Key Laboratory of Bioelectronics, Institute of Advanced Materials, Southeast University, 211189 Nanjing, China

**Keywords:** Liquid crystals, Actuators, Liquid crystals

## Abstract

In recent years, light-guided robotic soft actuators have attracted intense scientific attention and rapidly developed, although it still remains challenging to precisely and reversibly modulate the moving directions and shape morphing modes of soft actuators with ease of stimulating operation. Here we report a strategy of building a multi-stimuli-responsive liquid crystal elastomer soft actuator system capable of performing not only multi-directional movement, but also different shape morphing modes. This strategy is based on the selective stimulation of specific domains of the hierarchical structured actuator through the modulation of three wavelength bands (520, 808, 980 nm) of light stimulus, which release the actuation system from light scanning position/direction restriction. Three near-infrared dual-wavelength modulated actuators and one visible/infrared tri-wavelength modulated multi-directional walker robot are demonstrated in this work. These devices have broad application prospects in robotic and biomimetic technology.

## Abstract (web summary)

Light-guided robotic soft actuators have attracted intense scientific attention but it remains challenging to modulate the moving directions and shape morphing modes. Here the authors report a stimuli-responsive soft actuator system which is capable of performing multi-directional movement as well as different shape morphing modes.

## Introduction

In recent years, soft actuators as one kind of the most exciting and attractive soft matter materials, have emerged as a significant and extensive research area, and dramatically accelerated the development of biomimetic devices^1^, manipulators^2^, sensors, and robotic technology^3,4^, owing to their advantageous features, such as multiple degrees of freedom, strong adaptability to changing surroundings, inexpensive raw materials, diversiform actuations, and integration of multi-functionality, etc^5–7^. However, despite considerable progress in the research of stimuli-responsive soft actuators^8–10^, it still remains challenging to precisely and reversibly modulate the moving directions and even the shape morphing modes of soft actuators with robust macroscopic motions, fast responsive rates, and in particular ease of stimulating operation.

To control the moving directions of robotic soft actuators, light as a wireless remote control, is undoubtedly the most effective and predominant stimulus^11–14^. Up till now, most of light-guided robotic soft actuators could only execute one-way directional or two-way bidirectional moving^15–26^, few four-way (multi-directional) actuators relied on meticulously controlling the spatial distribution of light on the sample surfaces to reversibly modulate the moving directions of soft actuators. In another word, scientists had to precisely adjust the scanning position/direction/intensity of light, to induce the local asymmetric shape deformations of actuators and consequently realize the macroscopic directional moving changes, such as turning left and right, coming forth and back, etc. For example, to produce a bidirectional moving, researchers shined the laser light on the rear part of the actuator to generate thermal gradient stress and force it to come forward; and shined the laser light on the head part to force it to go backward. In another approach, scientists waved the laser from one side to the other side of the actuator to force it to go left, and waved the laser in the opposite direction to force the actuator to go right, etc. However, this commonly used light modulation strategy seriously lacks ease of operation, in particular when handling microscale robotic soft actuators. It is hard to imagine a scenario that doctors used an extremely thin beam of light, located it through skin tissues accurately on a micron-sized specific region of a microscale soft robot planted inside organisms, and hoped it would move along a designed pathway to perform medical treatments. To remove the operation and configuration restriction of light stimuli is an important and urgent subject in soft actuator research area.

The integration and modulation of diversified shape morphing modes (such as shrinking, bending, curling, etc.) within a same soft actuator, is another intriguing challenge. One effective strategy was to build multi-stimuli-responsive soft actuators with hierarchical architectures possessing different functionalities towards different stimuli^27–29^. For example, several multi-stimuli-responsive liquid crystal elastomer^30–63^ (LCE)-based bilayered soft actuators that could undergo bending and chiral twisting^64^, right-handed and left-handed helical curlings^65^ in response to ultraviolet (UV) and near-infrared (NIR) light stimuli, have been fabricated by incorporating azobenzene chromophores and NIR photothermal conversion dyes into the top-layer and bottom-layer LCE matrices respectively. Another similar strategy took advantage of the difference of shape deformations induced by photochemical conversion and photothermal conversion effects to modulate the deformation degrees of soft actuators^66,67^. Nonetheless, all the above light-modulated multi-motion-mode actuators must involve both the visible/infrared (vis/IR)-triggered photothermal-induced phase transition effects and UV-induced *trans*-*cis* isomerization of azobenzene chromophores. However, due to the very limited light penetration capability, UV-guided soft actuator systems have strict scanning direction restriction. Moreover, UV light can only efficiently drive the actuations of thin polymeric films, while the applications of several hundred-micrometer-thick soft actuators are severely plagued by the very slow UV-responsive rates^64,65^. Thus, exploration of UV-alternative light-guided multi-motion-mode soft actuator systems becomes the second objective of this work.

To address the above two challenges, we report in this manuscript a strategy of constructing a multi-stimuli-responsive hierarchical structured LCE soft actuator system capable of performing not only multi-directional moving, but also different shape morphing modes. This strategy is based on the selective stimulation of specific domains of the hierarchical structured actuator through the modulation of three-wavelength bands (520 nm vs. 808 nm vs. 980 nm) of visible/NIR light stimulus, which help the LCE actuation system free of light scanning position/direction restriction. Three NIR dual-wavelength modulated actuators including a two-way switch, a dual-motion-mode shape morpher and a two-way walker, and one vis/NIR tri-wavelength modulated multi-directional walker robot are demonstrated herein.

## Results

### Design and preparation protocol

The logic of this design is to build multiple independent and non-interfered photothermal conversion systems responding to different wavelength bands of light, in separate regions of a hierarchical structured LCE material. In such a design, the local asymmetric shape deformations of the LCE actuators will not be influenced by the varied scanning position/direction/intensity of light, but the photon energy absorption difference between different regions.

**Fig. 1:**
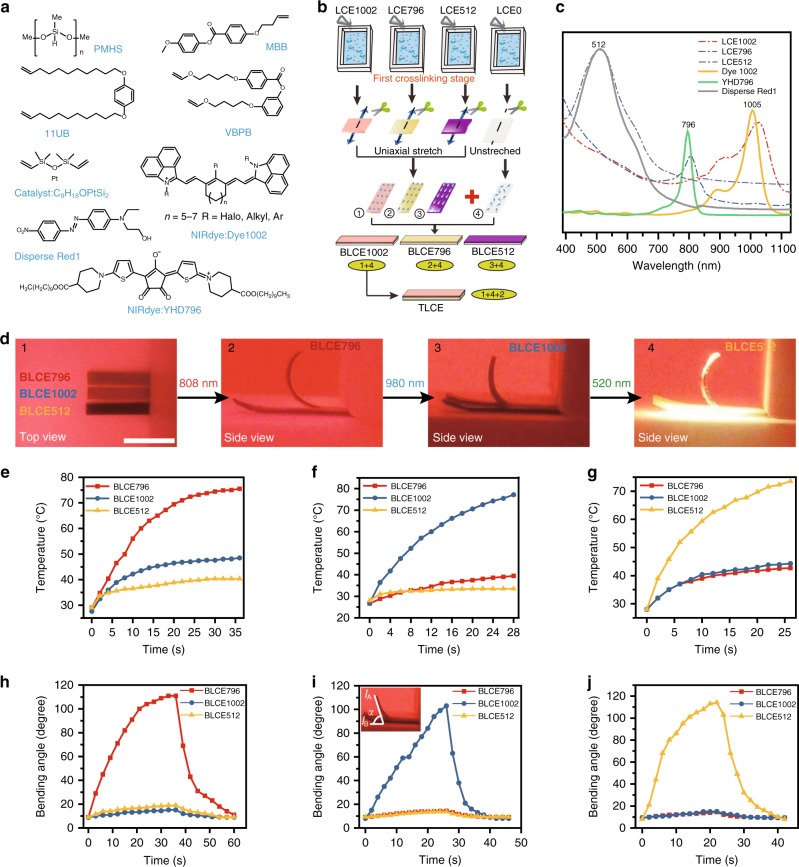
Design and investigation of NIR wavelength-selective responsive properties of LCE system. **a** The chemical components used in this LCE system. **b** Schematic illustration of the preparation procedures of BLCE1002, BLCE796, BLCE512 and TLCE. **c** UV-Vis-IR absorption spectra of YHD796 (conc. = 1.4 × 10^−3^ mg mL^−1^), Dye1002 (conc = 1.5 × 10^−3^ mg mL^−1^) and Disperse Red1 (conc = 5.0 × 10^−3^ mg mL^−1^) in dichloromethane, and the corresponding LCE796, LCE1002, and LCE512 films. **d** The photographs of the actuation motions of BLCE796, BLCE1002, and BLCE512 films with one end fixed, under the stimulation of 808 nm, 980 nm, and 520 nm light respectively (scale bar = 0.5 cm). Temperature profiles of BLCE796, BLCE1002, and BLCE512 films irradiated by (**e**) 808 nm, (**f**) 980 nm, and (**g**) 520 nm light. The diagrams of the included angle *α* of BLCE796, BLCE1002, and BLCE512 plotted against the illumination time of (**h**) 808 nm, (**i**) 980 nm, and (**j**) 520 nm light. Source data are provided as a Source Data file

The detailed chemical components used in this work are shown in Fig. 1a, we chose the classical polysiloxane-based LCE system including poly(methylhydrosiloxane) (PMHS), a mesogenic monomer 4-but-3-enyloxy-benzoic acid 4-methoxy-phenyl ester (MBB), Karstedt catalyst and two crosslinkers (11UB and VBPB) which were mixedly used for tuning the LC-to-isotropic phase transition temperature (*T*_i_) of the LCE material. Most importantly, three organic NIR absorbing dyes, YHD796^68^, commercially available Dye1002^15^ and Disperse Red1^69,70^ were selected as three independent photothermal conversion fillers, which had sharp and almost non-interfered absorptions (around 796 nm, 1005 nm, and 512 nm respectively) in the vis/IR region as shown in Fig. 1c.

The fabrication protocols of the LCE actuators are schematically illustrated in Fig. 1b. In general, four kinds of fundamental LCE films, LCE1002, LCE796, LCE512, and LCE0 were prepared. The LCE1002, LCE796, and LCE512 films, designated as the photo-responsive functional parts, were uniaxial-stretched monodomain LCE films doped with three organic dyes (Dye1002, 0.03 wt%; YHD796, 0.03 wt%; Disperse Red1, 0.11 wt%) respectively, whereas LCE0 was a polydomain LCE film without mechanical alignment nor incorporation of any photothermal conversion fillers. Meanwhile, the mole percentages of two crosslinkers VBPB and 11UB in the three LCE matrices were different. In LCE0, we applied 8 mol% of VBPB and 2 mol% of 11UB as the crosslinkers; in LCE512, we used 10 mol% of 11UB solely; while LCE1002 and LCE796 systems had 1.7 mol% of VBPB and 8.3 mol% of 11UB instead. The addition of more VBPB was used for achieving a higher *T*_i_ (88.3 °C, Supplementary Fig. 1a) than those (74.5~71.5 °C, Supplementary Fig. 1b–d) of LCE796, LCE1002, and LCE512 films, to ensure that LCE0 film could preserve enough mechanical strength to support the actuator system when LCE1002 or LCE796 or LCE512 film was heated to above its *T*_i_. The dye-doped LCE1002, LCE796, and LCE512 films, compared with three neat organic dyes, had broader optical absorption peaks which slightly red-shifted to 806 nm, 1025 nm, and 513 nm respectively, as demonstrated in Fig. 1c.

With these four fundamental LCE films in hand, we could synthesize bilayer- or trilayer-structured LCE actuators by adopting the classical two-step hydrosilylation crosslinking method^71^. As shown in Fig. 1b, the corresponding chemical reagents of LCE1002, LCE796, LCE512, and LCE0 dissolved in toluene were poured into four polytetrafluoroethylene (PTFE) rectangular molds (4.0 cm long × 2.0 cm wide × 1.5 cm deep) respectively, followed by ultrasonic treatments for 1.5 min. After heating in an oven at 60 °C for 2 h, these pre-crosslinked LCE films were removed from molds, and then cut into ribbons, dried for several hours. The pre-crosslinked LCE1002, LCE796, and LCE512 strips were uniaxially stretched along the longitudinal direction to obtain a monodomain alignment of mesogens, whereas LCE0 was used without any mechanical treatment so that its LC molecules remained in polydomain state as shown in Supplementary Fig. 2. Subsequently, the pre-crosslinked LCE1002 or LCE796 or LCE512 strip was stuck on the top of the pre-crosslinked LCE0 film, to provide bilayered LCE material (named as BLCE1002 or BLCE796 or BLCE512). The trilayered LCE material (TLCE) was prepared by stamping one LCE796 strip on one side, and one LCE1002 strip on the reverse side of the pre-crosslinked LCE0 film. Eventually, the bilayer- or trilayer-structured LCE material would be formed through a further heating in the oven at 60 °C for 2 days to complete the second-step hydrosilylation crosslinking procedure, which could not only lock the monodomain/polydomain mesogenic aligment of each LCE layer, but also spontaneously glue all the LCE layers together due to the covalent bonding of residual unreacted vinyl groups and Si–H groups on the interfaces of the pre-crosslinked LCE samples^64^, as demonstrated in Supplementary Fig. 3. The thickness of the TLCE film was ca. 500 μm (Supplementary Fig. 4).

### Vis/IR wavelength-selective response

The vis/IR wavelength-selective responsive behavior of this LCE system was first investigated. As shown in Fig. 1d, BLCE796, BLCE1002, and BLCE512 films were placed side by side on the table, the right end of each sample was fixed, and vis/NIR light was illuminated on both samples at the same time. Under the irradiation of 808 nm NIR light (0.18 W cm^−2^), the temperature of BLCE796 film quickly jumped to 74.5 °C in 32 s (Fig. 1e), which exceeded the *T*_i_ of the upper LCE796 layer, and triggered the upward bending of the BLCE796 film, whereas the temperatures of BLCE1002 and BLCE512 films reached to ca. 48 °C and 40 °C only. On the contrary, when exposed to 980 nm NIR light (0.19 W cm^−2^), the temperature of BLCE1002 film quickly rose to above the *T*_i_ (74.5 °C) of the upper LCE1002 layer in 26 s, which induced the macroscopic upward bending motion of BLCE1002 film, while BLCE796 and BLCE512 films kept almost motionless since their surface temperatures were slightly raised to around 39 °C and 33 °C, as shown in Fig. 1f. Compared with YHD796 and Dye1002, Disperse Red1 had much weaker photothermal conversion efficiency, thus the concentration of Disperse Red1 in LCE512 was increased from 0.03 to 0.11 wt%. As shown in Fig. 1g and Supplementary Movie 1, under the illumination of 520 nm light (47 mW cm^−2^) for 23 s, the surface temperatures of BLCE1002, BLCE796, and BLCE512 films appeared as 44 °C, 42 °C, and 71.5 °C, which only caused the upward bending motion of BLCE512 film. The photo-responsive bending rates of BLCE796, BLCE1002, and BLCE512 films were evaluated by recording the included angle *α* between line *l*_A_ (tangent line to the left endpoint of the arc bending sample) and the horizontal line *l*_B_, against the light illumination time, as plotted in Fig. 1h–j. It could been seen that all BLCE796, BLCE1002, and BLCE512 films performed a continuous bending motion, and achieved their maximum bending angles at 111°, 103°, and 115°, after 33 s stimulation of 808 nm NIR light, 26 s stimulation of 980 nm IR light, and 23 s stimulation of 520 nm visible light respectively.

### Two-way soft actuator demonstrations

**Fig. 2:**
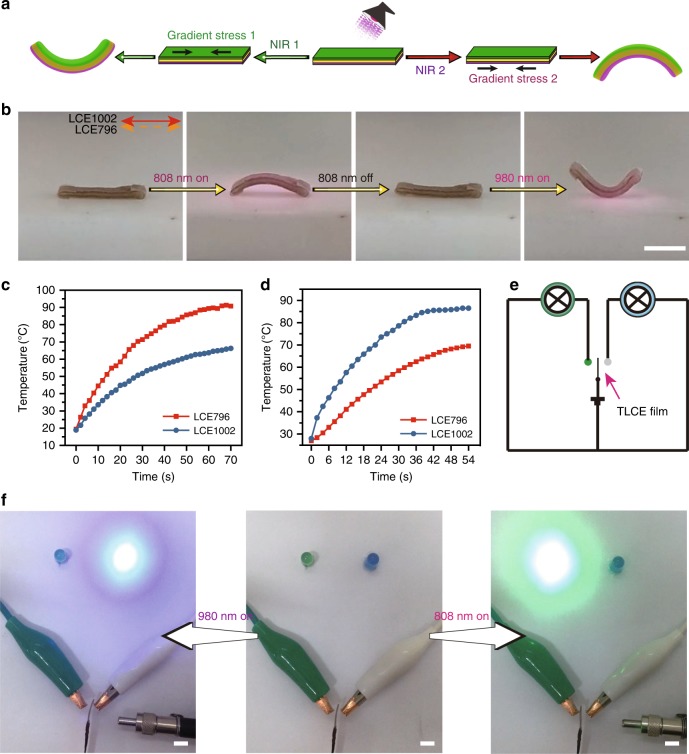
NIR dual-wavelength modulated two-way switch. **a** Schematic illustration and **b** the real image records of NIR dual-wavelength-selective shape deformations of a TLCE film (scale bar = 0.5 cm). The diagrams of the surface temperatures of the bottom LCE796 and upper LCE1002 layers of the TLCE sample plotted against the illumination time of **c** 808 nm and **d** 980 nm NIR light. **e** Circuit diagram of the designed two-way switch device. **f** Photographs of a TLCE film coated with conductive material acting as a two-way switch under the control of 808 nm and 980 nm NIR light. Source data are provided as a Source Data file

Encouraged by the vis/IR wavelength-selective actuation results, we started to use this hierarchical structured LCE system to fabricate diverse multi-wavelength modulated actuators. The first example was a two-way switch based on a trilayer-structured LCE material. As schematically illustrated in Fig. 2a, when a TLCE actuator was stimulated by one NIR light (808 nm or 980 nm), either the corresponding longitudinally aligned LCE796 or LCE1002 layer would respond and shrink when the photothermal energy was accumulated enough to raise its temperature to above the *T*_i_, whereas the middle LCE0 layer would not actuate since its mesogens were arranged in a polydomain manner. Meanwhile, LCE0 with low thermal conductivity (ca. 0.28 W m^−1^ K^−1^, measured by using the laser flash analysis method^72^, Supplementary Fig. 5) acted as a soft thermal insulating layer preventing the transmission of the heat generated from the top layer to the bottom layer, to some extent, so that the bottom LCE796 or LCE1002 layer could not obtain enough thermal energy to trigger the LC-to-isotropic transition-induced actuation, and the soft thermal insulating layer thickness should be at least twice of the surface layer thickness, as demonstrated in Supplementary Figs. 6 and 7. Overall, the gradient stress generated between the top layer and middle/bottom layers would induced upwards/downwards bending.

A demonstration was shown in Fig. 2b and Supplementary Movie 2, such a TLCE film could be stimulated by two NIR sources to execute opposite bending modes. Under the irradiation of 808 nm NIR light, TLCE film would bulge and bend downwards, and further recover back to the original linear structure after the removal of NIR light. When the same TLCE film was scanned by 980 nm NIR light, an opposite upward bending was found. The surface temperature variations of the bottom LCE796 and top LCE1002 layers of TLCE film during the two NIR light irradiation process were further measured by using a thermal imager instrument (FLUKE Ti90) which always showed the highest temperature, and a thermocouple thermometer (CEM DT-610B) which was adopted to detect the temperature of the low-temperature layer at the same time. As indicated in Fig. 2c,d, under the irradiation of 808 nm NIR light (0.18 W cm^−2^), the surface temperature of the bottom LCE796 layer was always increasing more quickly than that of LCE1002 layer, reached to its *T*_i_ (74.5 °C) in 33 s and stabilized at a saturated temperature of ca. 91 °C, whereas the surface temperature of the upper LCE1002 layer could just rise to ~66 °C. When stimulated by 980 nm NIR light (0.19 W cm^−2^), the surface temperature of LCE1002 layer was always higher than that of LCE796 layer, reached to its *T*_i_ (74.5 °C) in 26 s and stabilized at ca. 87 °C while the LCE796 layer could only go up to 69 °C.

A circuit of two light-emitting diode (LED) lights, using this TLCE film coated with conductive material as a two-way switch control, is schematically illustrated in Fig. 2e. Stimulating the TLCE switch with 980 nm NIR light, led to a rightward bending, and closed the right circuit to turn on the blue light. After the removal of 980 nm NIR light, the rightward bending switch recovered back to its original state, the right circuit opened and the blue light went out. A subsequent stimulation of 808 nm NIR light forced the TLCE switch to bend leftwards, closed the left circuit to turn on the green light. After the removal of 808 nm NIR light, the left circuit opened and the green light was turned off, as shown in Fig. 2f and Supplementary Movie 3.

**Fig. 3:**
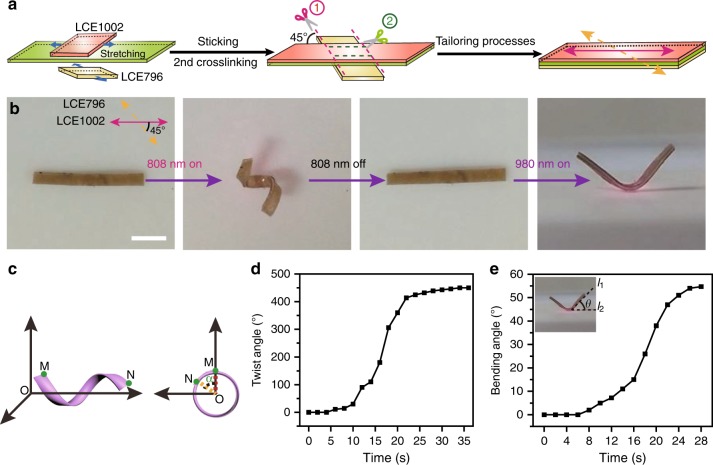
NIR dual-wavelength modulated dual-motion-mode shape morpher. **a** Schematic illustration of the preparation procedures of TLCE with a crossed angle of 45° between the alignment directions of the bottom LCE796 layer and top LCE1002 film. **b** The photographs of the actuating motions of TLCE under the illumination of 808 nm and 980 nm NIR light respectively (scale bar = 0.5 cm). **c** Schematic definition of the twist angle *α*. Twist angle *α* and included angle *θ* of TLCE plotted against the illumination time of **d** 808 nm and **e** 980 nm NIR light, respectively. Source data are provided as a Source Data file

The second example was a dual-motion-mode shape morpher. As demonstrated in Fig. 3a, a TLCE actuator was fabricated with a crossed angle of 45° between the alignment directions of LCE796 and LCE1002. Such a TLCE actuator could execute a right-handed helical twisting when it was irradiated by 808 nm NIR light and preform a bending motion under the stimulation of 980 nm NIR light respectively, as shown in Fig. 3b and Supplementary movie 4. To quantitatively characterize the helical rotation and bending motions of this TLCE ribbon, the twist angle *α* which was defined as a 360° rotation between line OM and line ON of the ribbon (Fig. 3c), and the bending angle *θ* which was defined as the angle between line *l*_1_ (tangent line to the right endpoint of the arc bending sample) and horizontal line *l*_2_, were measured respectively, as presented in Fig. 3d, e. It could be seen that the twist angle *α* increased slowly during the beginning 4 s, then grew rapidly in the next 29 s, and eventually achieved a maximum angle *α* of 450° under the stimulation of 808 nm NIR light (0.18 W cm^−2^). When exposed to 980 nm NIR light (0.19 W cm^−2^), the bending angle *θ* kept almost constant in the original 7 s, and then sharply increased to ca. 54^o^ in the next 20 s. Compared with the previously reported dual-motion-mode shape morphers built on azobenzene LCE system which required tens of minutes for actuating^64,65,73^, this TLCE ribbon could efficiently perform both motion modes in seconds timescale.

**Fig. 4:**
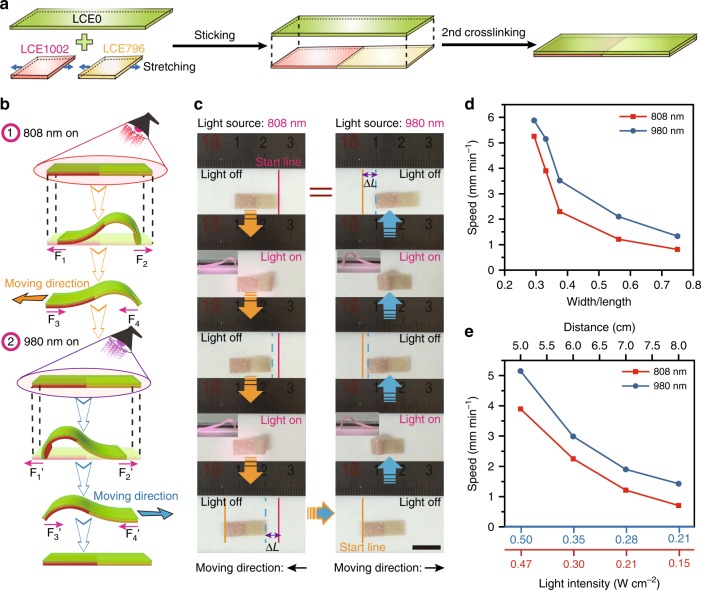
NIR dual-wavelength modulated two-way inchworm-like walker. **a** Schematic illustration of the fabrication of a two-way inchworm-like bilayered LCE walker. **b** Diagrammatic drawing of the force analysis for inchworm-like walker under the on-off stimulation of 808 nm and 980 nm NIR light. **c** Photographs showing the two-way inchworm-like walker moving left and right upon on-off irradiation of 808 nm and 980 nm NIR light (scale bar = 1.0 cm). The speed diagrams of the inchworm-like walker plotted against **d** varied width/length ratios (length = 16 mm) and **e** varied light intensities (length = 16 mm, *W/L* ratio = 0.33). Source data are provided as a Source Data file

The third example was a two-way inchworm-mimic walker capable of performing reversible bidirectional moving. The fabrication design is schematically illustrated in Fig. 4a, the two-way walker was a bilayered LCE film of which the first layer was a LCE0 ribbon and the second layer was a head-to-head joint of LCE1002 and LCE796 ribbons with both alignment directions parallel to the ribbon’s longitudinal orientation. Under the stimulation of 980 or 808 nm NIR light, the inchworm-like walker could selectively undergo asymmetric downward bending as shown in Fig. 4b. When stimulated by 808 nm NIR light, the right BLCE796 part acted as the inchworm body, bulged and bent downwards, whereas the left BLCE1002 part acting as the inchworm head, was passively dragged towards the BLCE796 part. Although, both BLCE1002 and BLCE796 parts moved towards the center, the asymmetric shape deformation provided the BLCE796 part a larger inclination which was fundamental in producing moving result^74^. According to Amontons’ law^75^, the friction force *F* = *μN*, where *μ* is the friction coefficient, *N* is the normal force component. After the removal of 808 nm NIR light, the right BLCE796 part would obtain a bigger friction than that of BLCE1002 part (*F*_4_ > *F*_3_), and push the inchworm walker to move leftwards, because the bulged BLCE796 part had much larger normal force than the flat BLCE1002 part. Repeating the on/off cycles of 808 nm NIR light stimulation (7 s per cycle, including 3 s light on and 4 s light off), drove the inchworm-like walker to move leftwards at a speed rate of *V*_left_ = 3.90 mm min^−1^. Similarly, when the wavelength of NIR light was changed to 980 nm, the inchworm-like walker would move rightwards at a speed rate of *V*_right_ = 5.15 mm min^−1^, through repeating the on/off cycles of 980 nm NIR light stimulation (14 s per cycle, including 4 s light on and 10 s light off), as shown in Fig. 4c and Supplementary movie 5.

The width to length ratio (*W*/*L*) of the LCE ribbon and the stimulating light intensity were two crucial parameters influencing on the moving speed of the inchworm-like walker. As shown in Fig. 4d, when the light source was placed at a spot 5 cm away from the sample, the maximum moving speed of the 16-mm-long inchworm-like walker would decrease from *V*_left_ = 5.26 mm min^−1^ and *V*_right_ = 5.88 mm min^−1^ to *V*_left_ = 0.81 mm min^−1^ and *V*_right_ = 1.33 mm min^−1^, along with the *W*/*L* ratio increasing from 29% to 75%, under the stimulation of 808 and 980 nm NIR light respectively. It indicated that the actuator with smaller *W/L* ratio would obtain faster moving speed. Meanwhile, the moving speed of the inchworm-like walker was plotted in relation to the stimulating light intensity which could be simply tuned by varying the linear distance (*d*) between the sample and the light source, and accurately measured by using an optic power meter. As shown in Fig. 4e, the moving speed was observed to decrease almost linearly with the increasing of *d* value. At *d* = 5 cm, the speeds of *V*_left_ and *V*_right_ were 3.90 and 5.15 mm min^−1^, respectively, and decreased to 0.71 and 1.43 mm min^−1^ when the *d* value increased to 8 cm. Obviously, weaker light intensity would slower the moving speed of the two-way walker.

### Three-wavelength modulated multi-directional actuator

**Fig. 5:**
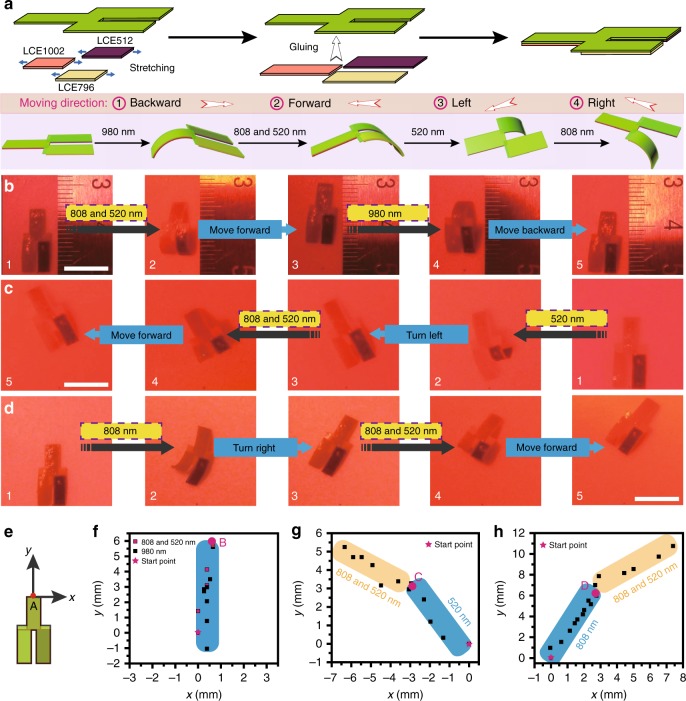
Photo-modulated multi-directional bilayered LCE walker. **a** Schematic illustration of the fabrication protocol and the moving mechanism of a multi-directional bilayered LCE walker. Photographs showing the multi-directional walker (**b**) moving either forward or backward upon on-off irradiation of 808/520 nm or 980 nm light, **c** moving left and then forward upon on-off irradiation of 520 nm and then 808/520 nm light, **d** moving right and then forward upon on-off irradiation of 808 nm and then 808/520 nm light. **e** The definition of the starting position coordinate of the LCE walker in *x–y* plane. The real-time position coordinate of the midpoint A of the LCE walker recorded in **f** moving forward and backward manner, **g** moving left and then forward manner, **h** moving right and then forward manner. Scale bar = 1.0 cm. Source data are provided as a Source Data file

Eventually, we prepared a vis/IR three-wavelength modulated multi-directional walker robot, as schematically illustrated in Fig. 5a. The multi-directional walker was a bilayered LCE film of which the top layer was a Y-shaped three-legged LCE0 film, and the bottom layer was consisted of LCE1002, LCE796, and LCE512 ribbons which were glued onto one of the three legs each and assigned as the front leg, left rear leg, and right rear leg respectively. Under the stimulation of 980 nm NIR light, the front leg of the walker could selectively undergo asymmetric downward bending and forced the three-legged robot to move backward. When stimulated by 808 and 520 nm light simultaneously, the two rear legs of the walker bulged together, bent downwards and pushed the three-legged robot to move forward. If either 808 nm NIR light or 520 nm green light was irradiated on the robot, only one rear leg would perform downward bending and make the walker turn to the opposite direction. Figure 5b–d and Supplementary movie 6 exhibited the vivid multi-directional moving scenario of this three-legged walker robot.

To investigate the robot moving statistically, the real-time position coordinate of the midpoint A of the three-legged walker under light stimulation (Supplementary movie 6) was analyzed by Tracker software, and plotted in Fig. 5e–h. We set (0, 0) in *x–y* plane as the starting point of the midpoint A of the LCE walker, and recorded its position coordinate once after every on/off light illumination cycle. It could be seen in Fig. 5f that when the walker robot was stimulated by the repeating on/off illumination cycles of either simultaneous 808/520 nm light (40 s per cycle, including 12 s light on and 28 s light off) or 980 nm NIR light (81 s per cycle, including 60 s light on and 21 s light off), the location distribution of the midpoint A of the robot in one round trip was almost in a straight line. When exposed to the repeating on/off illumination cycles of 520 nm light (34 s per cycle, including 15 s light on and 19 s light off), the midpoint A would first turn left-forward to position C, whereat the walker could continue to move forward after the light source was replaced with both 808 and 520 nm light (Fig. 5g). Similarly, when the wavelength of light was changed to 808 nm (27 s per cycle, including 7 s light on and 20 s light off), the walker robot would first move right-forward to position D, followed by moving forward in a straight line under the repeating on/off illumination cycles of 808 and 520 nm light simultaneously (Fig. 5h).

**Fig. 6:**
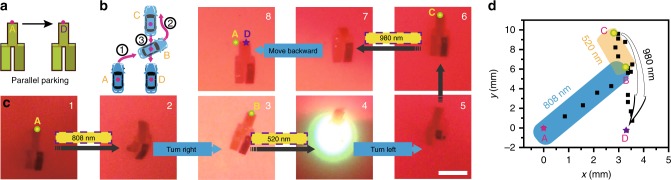
Photo-guided parallel parking of LCE walker robot. Schematic illustration of **a** the parallel parking of a vehicle and **b** how a vehicle accomplishes such a horizontal movement. **c** Photographs showing the process of such a vehicle-like walker robot moving horizontally from position A to position D under the stimulation of 808 nm, 520 nm, and 980 nm light (scale bar = 1.0 cm). **d** The real-time position coordinate of the midpoint A of this LCE walker recorded in parallel parking manner. Source data are provided as a Source Data file

In addition, the walker robot was able to realize more complex moving mode, such as the parallel parking of a vehicle as schematically illustrated in Fig. 6a, b. It could be seen from Fig. 6c, d and Supplementary movie 7 that the walker robot could move horizontally from position A to the parallel right position D in three steps: (1) moving right and forward to position B under the repeating on/off illumination cycles of 808 nm light; (2) turning left and forward from position B to position C under the repeating on/off illumination cycles of 520 nm light; (3) reversing backward from position C to the destination D under the repeating on/off illumination cycles of 980 nm light. These experiments demonstrated that the modulation of the wavelength bands of light stimulus could effectively control the multi-directional moving of such a vehicle-like LCE walker robot.

## Discussion

In conclusion, we describe in this manuscript a series of multi-wavelength modulated soft actuators capable of performing reversible multi-directional moving and dual-motion-mode shape morphing. Integration of multiple independent and non-interfered photothermal conversion systems in a hierarchical structured LCE material, would generate gradient stress inside the LCE matrices and induce the consequent asymmetric shape deformations of the macroscopic actuators, under the stimulation of different wavelengths of light.

This multi-stimuli-responsive actuator design has one obvious advantage: the multi-directional moving and dual-motion-mode shape morphing of these multi-wavelength modulated soft actuators are induced by the photon energy absorption difference between multiple independent and non-interfered photothermal conversion regions, and thus are free of light scanning position/direction restriction. For example, as shown in Fig. 2f, the two NIR light sources which were both set at the same spot, could effectively drive the TLCE switch to bend either leftwards or rightwards. The two walker robots are another striking example. As long as light was irradiating on the sample, the walkers could freely change the moving direction by just tuning the wavelength of the stimulating light, and required no further meticulous arrangement of the incident angle or scanning position of the incoming light source (Figs. 4c, 5b–d). Furthermore, since the vis/IR light stimuli possessed high tissue penetration capabilities, these light-guided robotic soft actuators have broad application prospects in biomedical technology.

We believe that this strategy could be further adopted to build four or even more independent photothermal conversion systems in hierarchical structured polymeric matrices to synthesize multi-wavelength modulated soft actuators with more functionalities, as long as we could find more organic photothermal conversion dyes which had non-interfered optical absorptions in the visible and infrared light regions. We hope this work would pave the way for multi-stimuli-responsive soft actuators with robust macroscopic motions, fast responsive rates and ease of stimulating operation.

## Methods

### General considerations

All the used reagents, starting materials and instrumentation descriptions are described in Supplementary Methods. The LC monomer MBB and the crosslinkers 11UB were synthesized according to the literature procedures, the synthetic protocol and NMR spectra of VBPB are included in Supplementary Figs. 8–10 and Supplementary Methods. Supplementary Movie 1 presents a side view of the actuation motions of BLCE1002, BLCE796, and BLCE512 films with one end fixed, under the stimulation of 980, 808, and 520 nm light respectively. Supplementary Movie 2 presents a side view of the shape deformations of TLCE film under the stimulation of 808 and 980 nm NIR light respectively. Supplementary Movie 3 presents a top view of the actuation motions of a two-way switch under the control of 808 and 980 nm NIR light. Supplementary Movie 4 presents a side and top view of a TLCE-based dual-motion-mode shape morpher under the control of 808 and 980 nm NIR light. Supplementary Movie 5 presents a side and top view of a two-way walker moving left and right upon on-off irradiation of 808 and 980 nm NIR light. Supplementary Movie 6 presents a top view of a multi-directional walker robot moving forward, backward, left, and right upon on-off irradiation of 808, 980, and 520 nm light. Supplementary Movie 7 presents a top view of a walker robot realizing parallel parking upon on-off irradiation of 808, 520, and 980 nm light.

### Preparation of the trilayered LCE material

In general, two pre-crosslinked LCE1002 and LCE796 films (2 cm long × 1 cm wide) were uniaxially stretched to ca. 130% of their original lengths. Then, the pre-crosslinked LCE1002 film was stamped on the top of a pre-crosslinked LCE0 film, whereas the pre-crosslinked LCE796 film was stuck on the reverse side of the pre-crosslinked LCE0 with a crossed angle of either 0° or 45° between the alignment directions of LCE796 and LCE1002. The trilayered LCE sample was placed on an oven and heated at 60 °C for 2 days to provide the fully crosslinked trilayered LCE membrane. Finally, the trilayered LCE film was tailored along the alignment direction of LCE1002 film into ribbons (16 mm long × 1.5 mm wide).

### Fabrication procedure of the two-way switch device

A TLCE film was placed horizontally on the table, followed by adding one drop (0.04 mL, 4 mg mL^−1^ dispersion in ethanol) of silver nanowires (AgNWs-L70, Nanjing XFNANO Materials Tech Co., Ltd) in ethanol onto the surface, and dried for 30 min in the ambient condition to evaporate residual ethanol. Subsequently, the TLCE film was carefully turned over to have one drop of silver nanowires in ethanol added onto the reverse surface, dried for another 30 min to give the desired two-way switch. The two-way switch experiment device was assembled by a TLCE-based two-way switch, two LED light and a Li-ion battery (KH168, 3.7 V) which were connected together by thin copper conductors.

### Preparation of the bilayered inchworm-like LCE walker

In general, a pre-crosslinked LCE796 (2 cm long × 1 cm wide) and a pre-crosslinked LCE1002 (2 cm long × 1 cm wide) were carefully glued together in a head-to-head manner that their alignment directions were parallel to each other, with the help of a rubber pressure-sensitive adhesive (purchased from SHUSHI GROUP CO., LTD.), and were further uniaxially stretched to ca. 140% of their original lengths. Subsequently, the unstretched polydomain LCE0 film was stamped on the top of the above head-to-head joint LCE796/LCE1002 film. The bilayered LCE material was placed on an oven and heated at 60 °C for 2 days to provide the fully crosslinked bilayered LCE membrane. Finally, the bilayered LCE film was tailored along the alignment direction of LCE796/LCE1002 film into ribbons, to fabricate inchworm-like LCE walkers.

### Preparation of the multi-directional LCE walker

In general, three fully crosslinked LCE films including a LCE796 (7 mm long × 2.5 mm wide), a LCE512 (7 mm long ×  2.5 mm wide) with a stretching ratio about 150% of their original lengths and a LCE1002 (7 mm long × 3 mm wide) with a stretching ratio ~140% of its original length, were carefully glued to a Y-shaped three-legged LCE0 film, by using a silicone adhesive (HJ-T326, Dongguan Fangguan Industrial Materials Co. Ltd.). Subsequently, the bilayered LCE film was kept at room temperature for 2 h to provide the desired multi-directional LCE walker.

## Supplementary information

Supplementary Information

Description of Additional Supplementary Files

Supplementary Movie 1

Supplementary Movie 2

Supplementary Movie 3

Supplementary Movie 4

Supplementary Movie 5

Supplementary Movie 6

Supplementary Movie 7

Source Data

## Data Availability

The source data underlying Figs. 1c, e–j, 2c, d, 3d, e, 4d, e, 5f–h, and 6d, Supplementary Figs. 1, 3c, f, 5, and 7b are provided as a Source Data file. The data underlying all figures in the main text and supplementary information are publicly available at 10.6084/m9.figshare.9860255.v1.
