# Supplementary material for: Visible and infrared three-wavelength modulated multi-directional actuators
Source: Nat Commun. 2019 Oct 4;10:4539. doi: 10.1038/s41467-019-12583-x (PMC6778143; doi:10.1038/s41467-019-12583-x)
Supplement: Supplementary file 1 — Supplementary Information [file 41467_2019_12583_MOESM1_ESM.pdf]

Supplementary Information

Visible and Infrared Three-Wavelength Modulated Multi-Directional Actuators

Zuo et al.

*School of Chemistry and Chemical Engineering, Jiangsu Province Hi-Tech Key Laboratory for Bio-medical Research, State Key Laboratory of Bioelectronics, Institute of Advanced Materials, Southeast University, Nanjing, 211189, China

*Correspondence and requests for materials should be addressed to H.Y. (email: yangh@seu.edu.cn).

Supplementary Figures

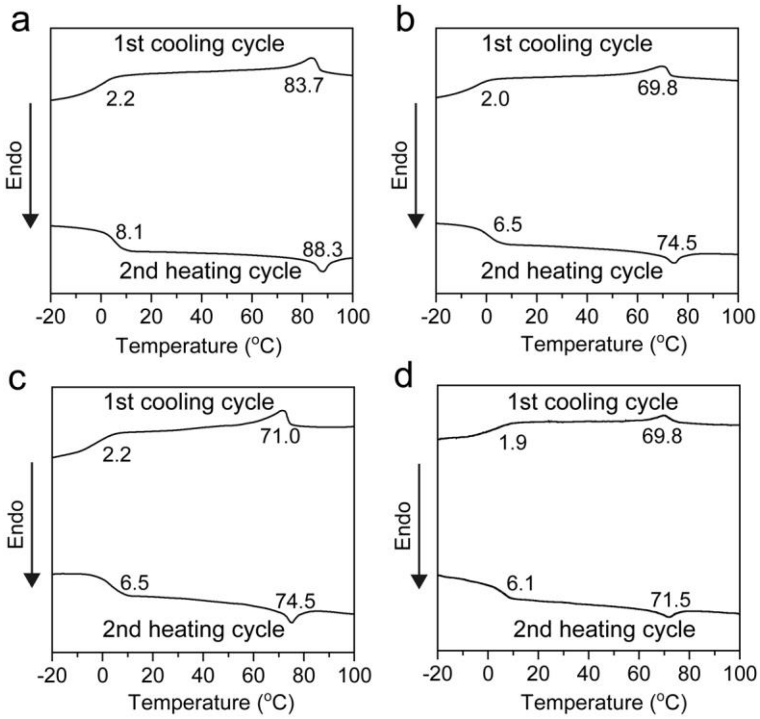

Supplementary Figure 1 |. DSC curves of three LCE films. DSC curves of (a) LCE0, (b) LCE796, (c) LCE1002 and (d) LCE512 during the first cooling and second heating scans at a rate of 10 ^{o}C/min under nitrogen atmosphere. Source data are provided as a Source Data file.

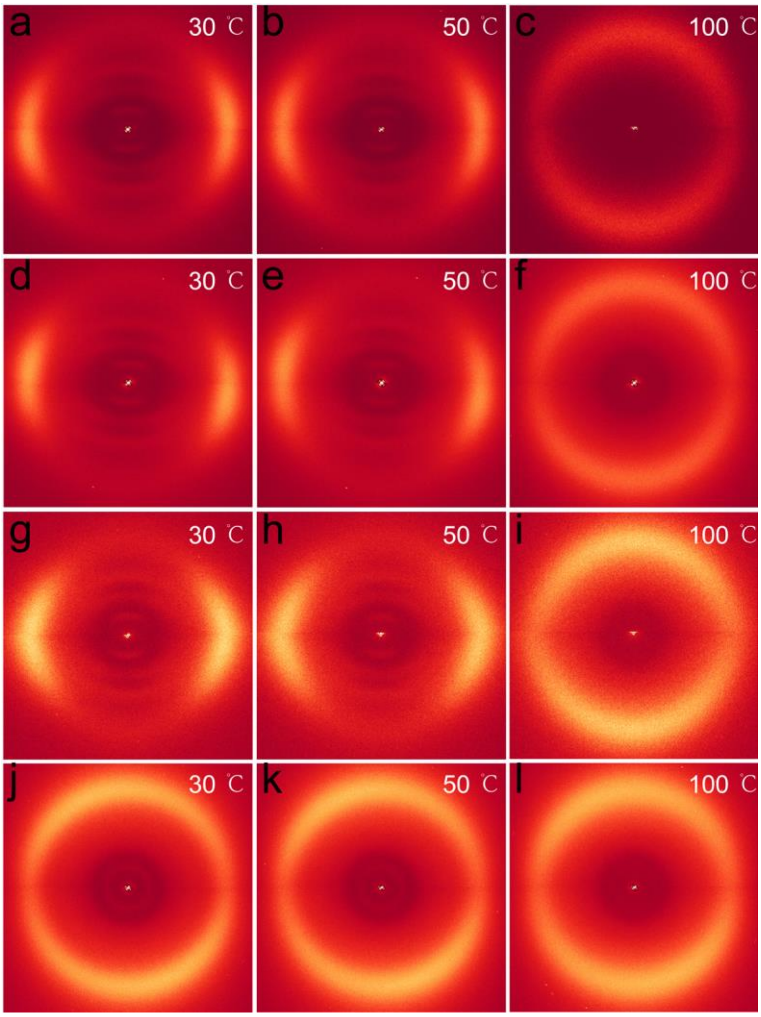

Supplementary Figure 2 |. Two-dimensional wide-angle X-ray diffraction patterns of three LCE films. The 2D WAXD patterns of (a-c) LCE796, (d-f) LCE1002, (g-i) LCE512 and (j-l) LCE0 measured at 30 ºC, 50 ºC and 100 ºC.

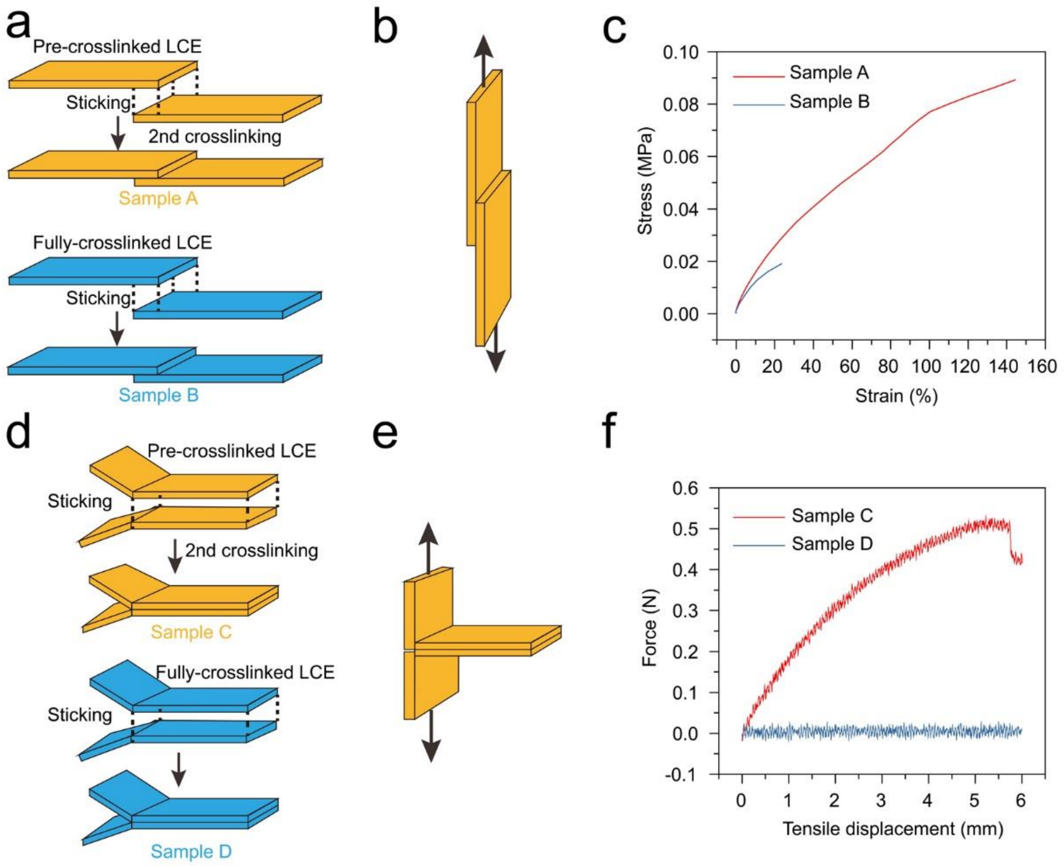

Supplementary Figure 3 |. Investigation of the stamped force between two overlap-pasted LCE layers. (a) Schematic illustration of the preparation procedures of sample A and sample B by sticking two pre-crosslinked LCE films and two fully-crosslinked LCE films respectively, in a dislocation overlap manner. (b) Schematic illustration of the shear strength test of samples A and B. (c) Stress-strain diagrams of samples A and B measured at 30 ^{o}C. (d) Schematic illustration of the preparation procedures of sample C and sample D by sticking two pre-crosslinked LCE films and two fully-crosslinked LCE films respectively, in a side-by-side manner. (e) Schematic illustration of the peel strength test of samples C and D. (f) Applied force versus tensile displacement diagrams of sample C and D measured at 30 ^{o}C. Source data are provided as a Source Data file.

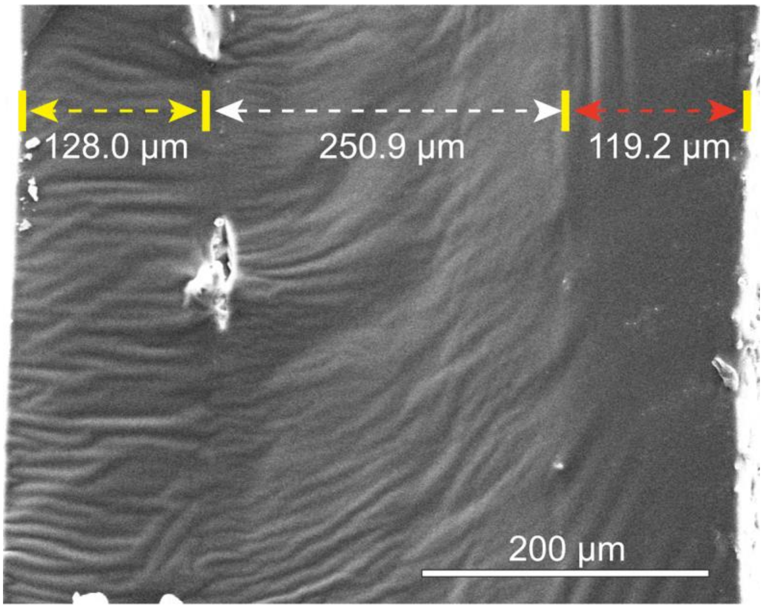

Supplementary Figure 4 |. SEM image of the cross-sectional area of TLCE film. SEM images showing the thickness of LCE1002, LCE0 and LCE796 (from left to right) of a TLCE film.

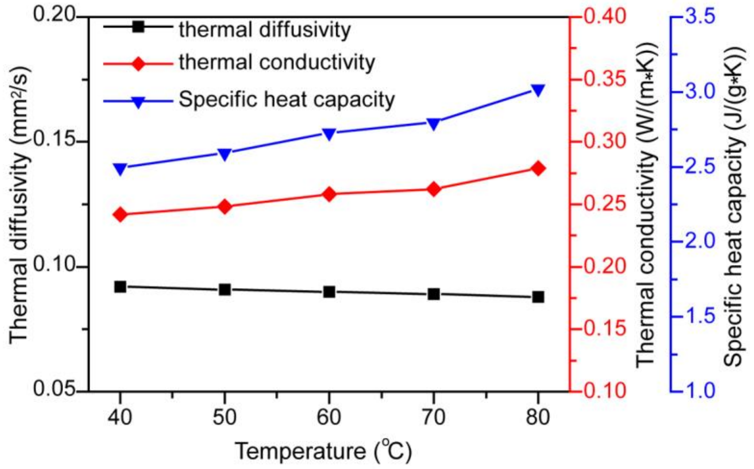

Supplementary Figure 5 |. Thermal conductive properties of LCE0. The thermal diffusivity, thermal conductivity and specific heat capacity properties of LCE0 versus temperature diagram. Source data are provided as a Source Data file.

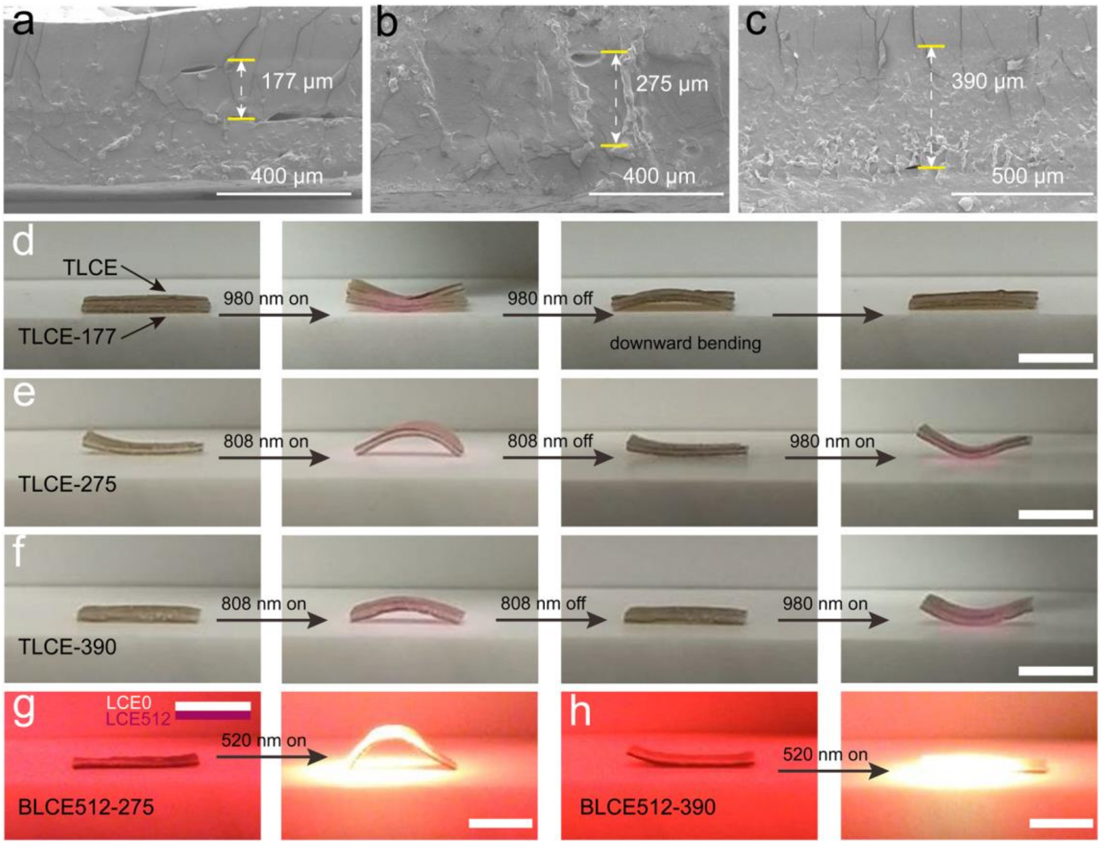

Supplementary Figure 6 |. The influence of the LCE0 layer thickness on the photo-actuation behaviors of the bilayered/trilayered actuators. SEM images of the cross-sectional areas of (a) TLCE-177, (b) TLCE-275 and (c) TLCE-390 films with different LCE0 layer thickness (177 μm, 275 μm and 390 μm respectively). The photographs of NIR dual-wavelength-selective shape deformations of (d) TLCE-177, (e) TLCE-275 and (f) TLCE-390 films. The photographs of the actuation behaviors of (g) BLCE512-275 (LCE0 layer thickenss: 275 μm) and (h) BLCE512-390 (LCE0 layer thickenss: 390 μm) under the stimulation of 520 nm. Scale bar = 0.5 cm.

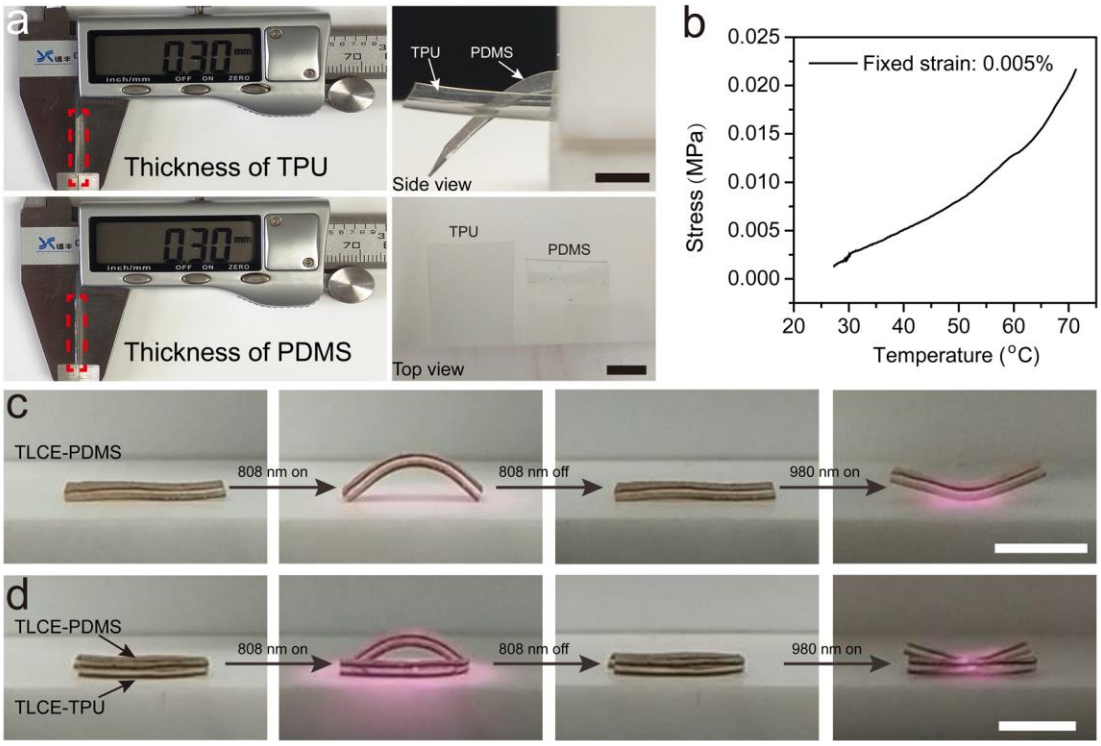

Supplementary Figure 7 |. The actuation behaviors of TLCE films using polydimethylsiloxane or polyurethane as the passive layer. (a) The softness comparison experiment of PDMS and TPU films (thicknesses ca. 0.3 mm). (b) Isostrain measurements of a monodomain LCE film at varied temperatures. (c,d) The photographs of NIR dual-wavelength-selective shape deformations of TLCE-PDMS and TLCE-TPU actuators. Scale bar = 0.5 cm. Source data are provided as a Source Data file.

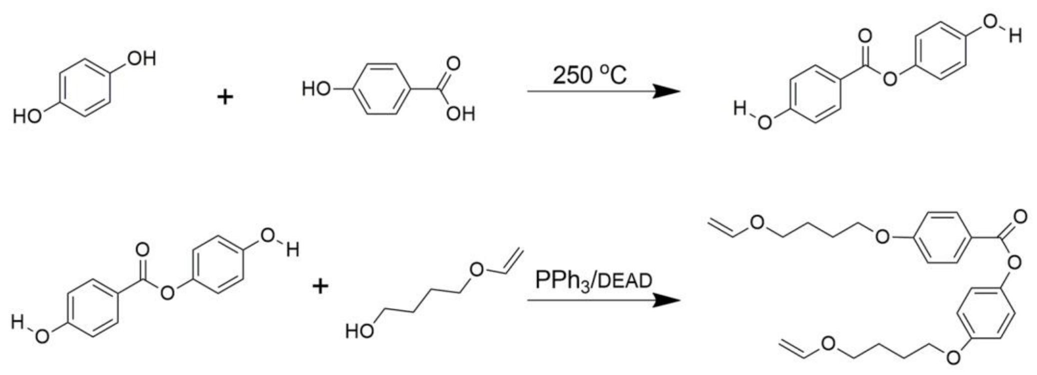

Supplementary Figure 8 |. Synthetic route of 4-[4-(vinyloxy)butoxy]phenyl 4-[4-(vinyloxy)-butoxy]benzoate (VBPB).

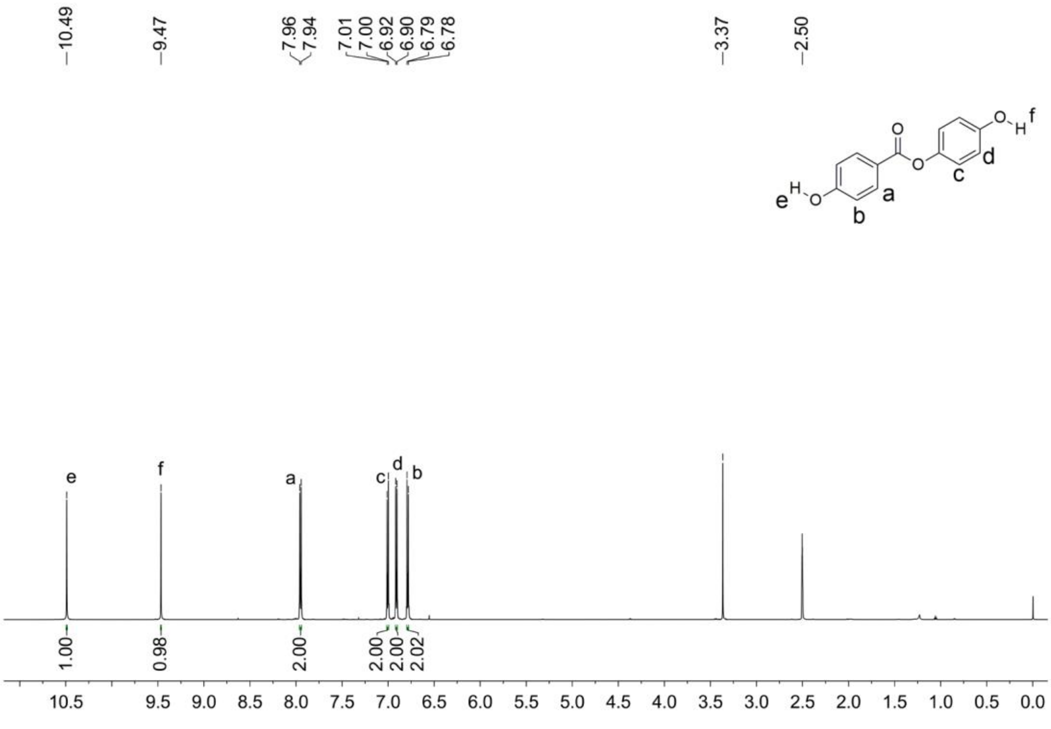

Supplementary Figure 9 |. ^{1}H NMR spectrum of 4-hydroxyphenyl 4-hydroxybenzoate.

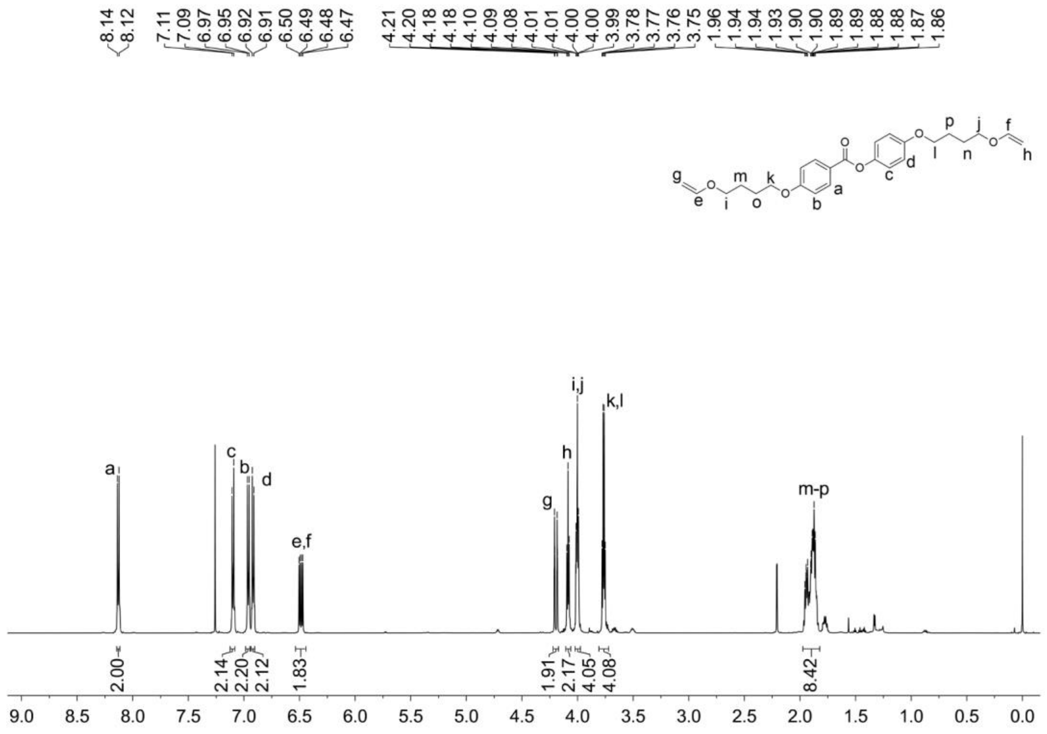

Supplementary Figure 10 |. ^{1}H NMR spectrum of 4-[4-(vinyloxy)butoxy]phenyl 4-[4-(vinyloxy)-butoxy]benzoate (VBPB).

Supplementary Methods

General Considerations. The NIR absorbing dye (Dye1002) was bought from QCR Solutions Corp (America). Disperse Red1 was bought from Aldrich Inc. Polymethylhydrosiloxanes (HMS-993, M.W. 2200-2400) were purchased from Gelest Inc. The PDMS film (BD Film KRN-300) was bought from Bald Advanced Materials Inc. and commercial TPU was bought from Xinrui insulating plastic materials Inc. 4-Hydroxybenzoic acid, hydroquinone, triphenylphosphine and other chemical reagents were purchased from Aladdin Inc. Toluene and tetrahydrofuran (THF) were redistilled from sodium and sodium benzophenone ketyl under nitrogen, respectively. All the chemical reactions were conducted in oven-dried glasswares under water-free and oxygen-free conditions.

Ultraviolet and visible and near-infrared spectra (UV-vis-NIR) of YHD796, Dye1002, Disperse Red1 and LCE films were recorded on LAMBDA950 (Perkin Elmer, America). Optical images and movies were recorded using camera phones. The scanning electron microscope (SEM) images were recorded on an Inspect F50 S3 field emission scanning electron microscope (FEI-SEM, America). Differential scanning calorimetry (DSC) spectra were measured on a TA Q100 instrument (New Castle, DE) under nitrogen purge at a heating/cooling rate of 10 °C/min. ^{1}H NMR spectra were recorded at a Bruker HW600 MHz spectrometer (AVANCE AV-600). Two-dimensional (2D) WAXD experiments were performed using Anton Paar SAXSpoint 2.0. The thermal diffusivity, thermal conductivity and specific heat capacity of the samples were measured on a NETZSCH LFA 467 equipment. A

MW-GX-808 and a MW-GX-980 NIR instruments (Changchun Laser Optoelectronics Technology Co., Ltd., China) were used as two NIR light stimuli sources. A MW-GX-520 green light instrument (Changchun Laser Optoelectronics Technology Co., Ltd., China) was used as a visible light stimuli source. All light intensities were measured by an optic power meter (LP-3B, Beijing Wuke Photoelectric Technique Co., Ltd., China).

Synthesis of 4-hydroxyphenyl 4-hydroxybenzoate.

To a 500 mL three-necked, round-bottomed flask equipped with a reflux condenser, 4-hydroxybenzoic acid (10.00 g, 0.07 mol, 1.0 equiv.) and hydroquinone (79.70 g, 0.72 mol, 10.0 equiv.) were added. After heating at 250 ^{o}C for 4 h, the reaction mixture was poured into 1.30 L of water and then filtrated to give the crude white solid powder, which was recrystallized in 200 mL of ethanol/water (0.8:1 v/v) to provide 4-hydroxyphenyl 4-hydroxybenzoate (8.00 g, yield: 48%) as a white solid powder. ^{1}H NMR (600 MHz, DMSO-d_6): δ 10.49 (s, 1H), 9.47 (s, 1H), 7.96 (d, J=12 Hz, 2H), 7.01 (d, J=6 Hz, 2H), 6.92 (d, J=12 Hz, 2H), 6.79 (d, J=6 Hz, 2H).

Synthesis of 4-[4-(vinyloxy)butoxy]phenyl 4-[4-(vinyloxy)-butoxy]benzoate (VBPB).

4-Hydroxyphenyl 4-hydroxybenzoate (7.00 g, 0.03 mol, 1.0 equiv.) and triphenylphosphine (16.80 g, 0.06 mol, 2.1 equiv.) were dissolved in 100 mL of THF in a round-bottomed flask under nitrogen atmosphere. After cooling down to 0 ^{o}C,

4-(vinyloxy)butan-1-ol (7.40 g, 0.06 mol, 2.1 equiv.) was added, followed by a dropwise addition of aza-1,2-dicarboxylate (11.10 g, 0.06 mol, 2.1 equiv.). The reaction mixture was stirred at room temperature for 12 h, concentrated *via* rotovap, diluted by 150 mL of hexane/AcOEt (5:1, v/v) and filtered off the precipitates. The organic solution was further concentrated and purified by silica gel column chromatography with petroleum ether/dichloromethane (2:1, v/v) as the eluent to provide VBPB (6.0 g, yield: 46%) as a white solid. ^{1}H NMR (600 MHz, $CDCl_3$): δ 8.14 (d, J=12 Hz, 2H), 7.11 (d, J=12 Hz, 2H), 6.97 (d, J=12 Hz, 2H), 6.92 (d, J=6 Hz, 2H), 6.50 (dd, J=12, 6 Hz, 2H), 4.21 (m, 2H), 4.10 (t, J=6 Hz, 2H), 4.01 (m, 4H), 1.86-1.96 (m, 8H).

Preparation of pre-crosslinked LCE1002 film. PMHS (50.0 mg, 0.83 mmol Si-H groups), MBB (198.7 mg, 0.67 mmol), 11UB (28.6 mg, 0.069 mmol), VBPB (6.0 mg, 0.014 mmol), 25 μL CH_2Cl_2 solution containing 0.1 mg NIR dye 1002 and 5.0 μL Karstedt catalyst solution (Platinum(0)-1,3-divinyl-1,1,3,3-tetramethyldisiloxane complex solution in xylene, Pt ~2%) were dissolved in 2.5 mL toluene, following by ultrasonication for 1.5 min in a PTFE rectangular mould (4.0 cm long × 2.0 cm wide × 1.5 cm deep). After heating in an oven at 60 ^{o}C for 3 h, the pre-crosslinked LCE1002 film was carefully taken out and cut into a strip (2 cm long × 1 cm wide).

Preparation of pre-crosslinked LCE796 film. PMHS (50.0 mg, 0.83 mmol Si-H groups), MBB (198.7 mg, 0.67 mmol), 11UB (28.6 mg, 0.069 mmol), VBPB (6.0 mg, 0.014 mmol), 25 μL CH_2Cl_2 solution containing 0.1 mg YHD796 and 5.0 μL Karstedt

catalyst solution (Platinum(0)-1,3-divinyl-1,1,3,3-tetramethyldisiloxane complex solution in xylene, Pt ~2%) were dissolved in 2.5 mL toluene, following by ultrasonication for 1.5 min in a PTFE rectangular mould (4.0 cm long × 2.0 cm wide × 1.5 cm deep). After heating in an oven at 60 ^{o}C for 3 h, the pre-crosslinked LCE796 film was carefully taken out and cut into a strip (2 cm long × 1 cm wide).

Preparation of pre-crosslinked LCE512 film. PMHS (50.0 mg, 0.83 mmol Si-H groups), MBB (198.7 mg, 0.67 mmol), 11UB (34.5 mg, 0.083 mmol) were dissoved in 2 mL toluene. The mixture was cast into a PTFE rectangular mould (4.0 cm long × 2.5 cm wide × 1.5 cm deep). Then, 0.5 mL toluene solution containing 0.3 mg Disperse red1 and 5.0 μL Karstedt catalyst solution (Platinum(0)-1,3-divinyl-1,1,3,3-tetramethyldisiloxane complex solution in xylene, Pt ~2%) were added, followed by ultrasonication for 1.5 min. After heating in an oven at 60 ^{o}C for 4 h, the pre-crosslinked LCE512 film was carefully taken out and cut into a strip (2 cm long × 1 cm wide).

Preparation of pre-crosslinked LCE0 film. PMHS (44.0 mg, 0.73 mmol Si-H groups), MBB (174.8 mg, 0.59 mmol), 11UB (6.07 mg, 0.015 mmol), VBPB (25.0 mg, 0.059 mmol) and 5.0 μL Karstedt catalyst solution (Platinum(0)-1,3-divinyl-1,1,3,3-tetramethyldisiloxane complex solution in xylene, Pt ~2%) were dissolved in 2.0 mL toluene, following by ultrasonication for 1.5 min in a PTFE rectangular mould (4.0 cm long × 2.0 cm wide × 1.5 cm deep). After heating in

an oven at 60 ^{o}C for 2 h, the pre-crosslinked LCE0 film was carefully taken out and cut into a strip (4 cm long × 1 cm wide) without any stretching.

Investigation of the stamped force between two overlap-pasted LCE layers. Taking advantage of Finkelmann's two-step crosslinking strategy, all the LCE layers could be spontaneously glued together due to the covalent bonding of residual unreacted vinyl groups and Si-H groups on the interfaces of the pre-crosslinked LCE samples. To investigate the stamped force between two overlap-pasted LCE samples, we performed the shear strength and peel strength tests of the overlap-pasted LCE samples as illustrated in Supplementary Fig. 3.

For comparison purpose, we stuck two pre-crosslinked LCE films and two fully-crosslinked LCE films respectively in a dislocation overlap manner (samples A and B, Supplementary Fig. 3a), for shear strength test experiment (Supplementary Fig. 3b); and stuck two pre-crosslinked LCE films and two fully-crosslinked LCE films respectively in a side-by-side overlap manner (samples C and D, Supplementary Fig. 3d), for peel strength test experiment (Supplementary Fig. 3e). As shown in Supplementary Fig. 3c, the stress-strain measurements of the shear strengths were conducted along the longitudinal direction of the LCE films with a dynamic mechanical analyzer bearing a tension clamp for static stress-strain with 1 mN preload force at a strain rate of 0.5 N min^{-1}. At 30 ^{o}C, the maximum tensile strength and the elongation-at-break value of the bilayered LCE sample A were ca. 0.09 MPa and 144% respectively, while the corresponding values of sample B only reached ca. 0.02

MPa and 24%.

As shown in Supplementary Fig. 3f, the peel strength test was conducted in the normal direction of the LCE film on a MTS E42 tensile tester with a crosshead speed of 5.0 mm min^{-1} at 30 ºC. It could been seen that the adhered part of sample D could be easily separated from each other under a very small force (about 0.01 N), whereas the adhered part of sample C could not been peeled off even after the pulling force was increased to 0.51 N which did break the ribbon. These experiments demonstrated that the pre-crosslinked LCE layers could be spontaneously glued together in the help of the covalent bonding of residual unreacted vinyl groups and Si-H groups on the interfaces during the second cross-linking stage.

Influence of the LCE0 layer thickness on the photo-actuation behaviors of the bilayered/trilayered actuators. The thickness of each layer of the bilayer or trilayer actuators indeed has an important influence on the photo-actuating behaviors. Three LCE0 samples with different thicknesses (177 μm, 275 μm and 390 μm, Supplementary Fig. 6a-c) were synthesized to further fabricate the corresponding TLCE samples (named as TLCE-177, TLCE-275 and TLCE-390) and two BLCE512 actuators (named as BLCE512-275 and BLCE512-390).

As shown in Supplementary Fig. 6d, when the thickness of LCE0 layer was decreased to 177 μm, the corresponding TLCE-177 actuator could not efficiently perform upward bending under the illumination of 980 nm NIR light, meanwhile executed an unexpected downward bending after the removal of 980 nm light,

whereas the contrastive TLCE sample (LCE0 layer thickness: 250 μm) which is described in the manuscript, TLCE-275 (Supplementary Fig. 6e) and TLCE-390 (Supplementary Fig. 6f) actuators bearing thicker LCE0 layers could efficiently execute wavelength-selective bending modes and fully recover to the original flat state after removal of light stimulus. This experiment implied that a thin thermal insulating layer could not efficiently prevent the transmission of the heat generated from the top layer to the bottom layer. Concerning that the surface layer (LCE1002 or LCE796) thickness was ca. 120 μm, the optimal thermal insulating layer thickness should be at least twice of the surface layer thickness.

However, thin passive layer would benefit the photo-actuating performance of bilayered LCE actuators. As shown in Supplementary Fig. 6g,h, it was difficult for BLCE512-390 film with a 390 μm thick passive layer to bend under the stimulation of 520 nm light, whereas BLCE512-275 film with a thinner passive layer could execute photo-actuation efficiently. Concerning that the surface layer (LCE1002 or LCE796) thickness was ca. 120 μm, the optimal passive layer thickness should be no more than 2.5 times of the surface layer thickness.

Investigation of the middle thermal insulating passive layer using other amorphous, transparent and low-thermal conductivity polymers. As shown in Supplementary Fig. 7a, two TLCE actuators (named as TLCE-PDMS and TLCE-TPU) were fabricated by using either a soft polydimethylsiloxane (PDMS, thickness ca. 0.3 mm, BD Film KRN-300) film or a relatively harder thermoplastic polyurethane (TPU,

thickness ca. 0.3 mm, bought from Xinrui insulating plastic materials Inc.) film as the middle passive layer. As shown in Supplementary Fig. 7c,d, only the softer TLCE-PDMS could execute upward/downward bending, whereas TLCE-TPU could not been actuated because the monodomain LCE layer could only generate very small internal mechanical force (ca. 21 KPa, Supplementary Fig. 7b) which could not force the hard TPU layer to bend. Thus, only these very soft and low-thermal conductivity polymers can been used as the passive layer.
