# Supplementary material for: Visible and infrared three-wavelength modulated multi-directional actuators
Source: Nat Commun. 2019 Oct 4;10:4539. doi: 10.1038/s41467-019-12583-x (PMC6778143; doi:10.1038/s41467-019-12583-x)
Supplement: Supplementary file 2 — Description of Additional Supplementary Files [file 41467_2019_12583_MOESM2_ESM.pdf]

Description of Additional Supplementary Files

File Name: Supplementary Movie 1
Description: A side view of the actuation motions of BLCE1002, BLCE796 and BLCE512 films with one end fixed, under the stimulation of 980, 808 and 520 nm light respectively.

File Name: Supplementary Movie 2
Description: A side view of the shape deformations of TLCE film under the stimulation of 808 and 980 nm NIR light respectively.

File Name: Supplementary Movie 3
Description: A top view of the actuation motions of a two-way switch under the control of 808 and 980 nm NIR light

File Name: Supplementary Movie 4
Description: A side and top view of a TLCE-based dual-motion-mode shape morpher under the control of 808 and 980 nm NIR light.

File Name: Supplementary Movie 5
Description: "A side and top view of a two-way walker moving left and right upon on-off irradiation of 808 and 980 nm NIR light.

File Name: Supplementary Movie 6
Description: A top view of a multi-directional walker robot moving forward, backward, left and right upon on-off irradiation of 808, 980 and 520 nm light.

File Name: Supplementary Movie 7
Description: A top view of a walker robot realizing parallel parking upon on-off irradiation of 808, 520 and 980 nm light.
